# Clinical validation of the EndoPredict test in node-positive, chemotherapy-treated ER+/HER2− breast cancer patients: results from the GEICAM 9906 trial

**DOI:** 10.1186/bcr3642

**Published:** 2014-04-12

**Authors:** Miguel Martin, Jan C Brase, Lourdes Calvo, Kristin Krappmann, Manuel Ruiz-Borrego, Karin Fisch, Amparo Ruiz, Karsten E Weber, Blanca Munarriz, Christoph Petry, Cesar A Rodriguez, Ralf Kronenwett, Carmen Crespo, Emilio Alba, Eva Carrasco, Maribel Casas, Rosalia Caballero, Alvaro Rodriguez-Lescure

**Affiliations:** 1 Medical Oncology Department, Gregorio Marañon University General Hospital, Dr. Esquerdo, 46, 28007 Madrid, Spain; 2 Sividon Diagnostics GmbH, Nattermannallee, 1, 50829 Cologne, Germany; 3 Medical Oncology Department, A Coruña University Hospital Complex, As Xubias, 84, 15006 A Coruña, Spain; 4 Medical Oncology Department, Virgen del Rocio University Hospital, Av Manuel Siurot, 0, 41013 Seville, Spain; 5 Medical Oncology Department, Valencian Institute of Oncology, Carrer de l'Estrela, 3, 46007 Valencia, Spain; 6 Medical Oncology Department, La Fe University Hospital, Carrer d'Antonio Ferrandis, 46026 Valencia, Spain; 7 Medical Oncology Department, Salamanca University General Hospital, Paseo San Vicente, 88-182, 37007 Salamanca, Spain; 8 Medical Oncology Department, Ramon y Cajal University Hospital, Ctra. de Colmenar Viejo, km. 9,100, 28034 Madrid, Spain; 9 Medical Oncology Department, Virgen de la Victoria University Hospital, Campus de Teatinos, s/n, 29010 Malaga, Spain; 10 GEICAM (Spanish Breast Cancer Research Group), Madrid, Spain; 11 Medical Oncology Department, Elche University General Hospital, Cami de L'Almassera, 11, 03204 Elche, Alicante, Spain

## Abstract

**Introduction:**

EndoPredict (EP) is an RNA-based multigene test that predicts the likelihood of distant recurrence in patients with estrogen receptor-positive (ER+), human epidermal growth factor receptor 2–negative (HER2−) breast cancer (BC) who are being treated with adjuvant endocrine therapy. Herein we report the prospective-retrospective clinical validation of EP in the node-positive, chemotherapy-treated, ER+/HER2− BC patients in the GEICAM 9906 trial.

**Methods:**

The patients (*N* = 1,246) were treated either with six cycles of fluorouracil, epirubicin and cyclophosphamide (FEC) or with four cycles of FEC followed by eight weekly courses of paclitaxel (FEC-P), as well as with endocrine therapy if they had hormone receptor–positive disease. The patients were assigned to EP risk categories (low or high) according to prespecified cutoff levels. The primary endpoint in the clinical validation of EP was distant metastasis-free survival (MFS). Metastasis rates were estimated using the Kaplan-Meier method, and multivariate analysis was performed using Cox regression.

**Results:**

The molecular EP score and the combined molecular and clinical EPclin score were successfully determined in 555 ER+/HER2− tumors from the 800 available samples in the GEICAM 9906 trial. On the basis of the EP, 25% of patients (*n* = 141) were classified as low risk. MFS was 93% in the low-risk group and 70% in the high-risk group (absolute risk reduction = 23%, hazard ratio (HR) = 4.8, 95% confidence interval (CI) = 2.5 to 9.5; *P* < 0.0001). Multivariate analysis showed that, in this ER+/HER2− cohort, EP results are an independent prognostic parameter after adjustment for age, grade, lymph node status, tumor size, treatment arm, ER and progesterone receptor (PR) status and proliferation index (Ki67). Using the predefined EPclin score, 13% of patients (*n* = 74) were assigned to the low-risk group, who had excellent outcomes and no distant recurrence events (absolute risk reduction vs high-risk group = 28%; *P <* 0.0001). Furthermore, EP was prognostic in premenopausal patients (HR = 6.7, 95% CI = 2.4 to 18.3; *P =* 0.0002) and postmenopausal patients (HR = 3.3, 95% CI = 1.3 to 8.5; *P =* 0.0109). There were no statistically significant differences in MFS between treatment arms (FEC vs FEC-P) in either the high- or low-risk groups. The interaction test results between the chemotherapy arm and the EP score were not significant.

**Conclusions:**

EP is an independent prognostic parameter in node-positive, ER+/HER2− BC patients treated with adjuvant chemotherapy followed by hormone therapy. EP did not predict a greater efficacy of FEC-P compared to FEC alone.

## Introduction

Several prognostic multigene tests have been developed for estrogen receptor-positive (ER+) early breast cancer (BC) patients [1-7]. Large clinical validation studies have demonstrated that molecular assays are useful for stratifying patients into risk categories and helpful in making clinical treatment decisions in ER+/node-negative BC patients. Much less is known, however, about the prognostic performance of these tests in patients with axillary lymph node–positive disease. So far, only a few of these assays have been validated in large node-positive BC cohorts treated with endocrine or chemoendocrine treatment. For instance, the 21-gene recurrence score (RS) was initially established and validated in node-negative BC patients [1,7,8]. Later, the SWOG-8814 study demonstrated that RS was able to predict distant metastases in node-positive BC patients [9]. However, SWOG-8814 and other trials [10] demonstrated that putative low-risk patients have a considerable, sustained risk for distant metastases. Therefore, the question remains whether multigene assays can be used to (1) identify node-positive BC patients for who can safely be spared from undergoing chemotherapy and (2) tailor more intensive or novel drug-based treatment strategies in clinically high-risk cohorts. Additionally, none of the available tests has yet been validated to predict taxane efficacy [10-12].

The EndoPredict (EP) test has recently been introduced as an RNA-based multigene test to predict the likelihood of distant recurrence in ER-positive/HER2-negative (ER+/HER2−) BC patients treated with adjuvant endocrine therapy. The test is designed to be used in a decentralized setting in molecular pathology laboratories [6,13-15]. Training in the use of EP was noticeably different compared to other prognostic tests: node-negative and node-positive ER+/HER2− BC patients (*n* = 964) were included in the multigene algorithm design, and a combined score of EP, tumor size and nodal status (EPclin) was defined in the large training cohort. EP was subsequently validated in two randomized phase III trials (Austrian Breast and Colorectal Cancer Study Group trials ABCSG6 and ABCSG8; *n* > 1,700) that included postmenopausal node-negative and node-positive BC patients treated with endocrine therapy alone [6]. Subgroup analyses within the ABCSG validation studies indicated that EP and EPclin could be used to identify subgroups showing remarkable differences in 10-year distant recurrence rates in patients with node-negative and node-positive disease. Although the ABCSG6 and ABCSG8 studies demonstrated that EP results enabled the identification of a subgroup of node-positive BC patients with particularly good clinical outcomes, the performance of EP in chemotherapy-treated, node-positive patients has not been evaluated yet.

In this study, we validated the EP score in node-positive ER+/HER2− BC patients in the GEICAM 9906 trial, who were treated with adjuvant chemotherapy followed by hormone therapy. We also evaluated whether EP results could predict the efficacy of incorporating weekly paclitaxel into anthracycline-based regimens.

## Methods

### Patients and tumor samples

The patients included in this study participated in the GEICAM 9906 trial, a randomized phase III trial comparing two adjuvant chemotherapy regimens after BC surgery in 1,246 women with lymph node–positive disease [16,17]. The patients were treated either with six 21-day cycles of 5-fluorouracil, epirubicin and cyclophosphamide (FEC; control arm) or with four 21-day cycles of FEC followed by eight weekly courses of paclitaxel (FEC-P; experimental arm). Hormone receptor–positive patients received hormone treatment after chemotherapy. The median follow-up duration for the whole cohort was 8.7 years. Details of the study design and the patients’ characteristics have been reported previously [16]. The study was performed in accordance with the Declaration of Helsinki and approved by the ethics committees at all participating institutions (Additional file 1: Figure S1) as well as the Spanish Health Authority. It is registered with the US National Institutes of Health (ClinicalTrials.gov Identifier: NCT00129922). All patients provided their written informed consent for therapy randomization and molecular analyses.

Tumor blocks for the EP validation were collected at the time of surgery. Formalin-fixed, paraffin-embedded (FFPE) tumor blocks were available from 800 of the 1,246 patients who participated in the GEICAM 9906 trial. Hematoxylin and eosin–stained sections from each FFPE tissue block were evaluated by a pathologist at a GEICAM central laboratory. ER, PR and Ki67 expression were assessed by immunohistochemistry (IHC) in a central laboratory. ER and PR staining were scored according to the Allred method as previously described [17,18]. Two 5-μm tissue sections were obtained from each tumor sample.

### RNA extraction and gene expression analysis

Total RNA was extracted from 5-μm whole FFPE tissue sections using a silica bead–based, fully automated isolation method (VERSANT Tissue Preparation Reagents Kit; Siemens Healthcare Diagnostics, Tarrytown, NY, USA) [19-21]. The method includes fully automated deparaffinization, DNase I digestion and an RNA extraction step. DNA-free total RNA from one FFPE section was ultimately eluted with 100 μl of elution buffer and stored at −80°C. To identify ER+/HER2− patients, *ESR1* and *ERBB2* gene expression levels were analyzed by qRT-PCR in the FFPE tumor samples, and predefined cutoff levels were applied as recently described [6]. A high concordance between quantitative RT-PCR and IHC-based assessments of ER/HER2 status has been reported before [21]. The EP assay is based on the quantification of eight cancer-related genes of interest (*BIRC5, UBE2C, DHCR7, RBBP8, IL6ST, AZGP1, MGP and STC2*) and three reference genes (*CALM2, OAZ1 and RPL37A*). This assay was performed as previously described [6]. In brief, samples were measured in triplicate in 384-well plates by quantitative RT-PCR using an ABI PRISM 7900HT Sequence Detection System (Applied Biosystems, Foster City, CA, USA) and the SuperScript III Platinum One-Step qRT-PCR Kit with ROX (Invitrogen, Karlsruhe, Germany). The thermal protocol included 30 minutes at 50°C, 20.5 minutes at 8°C and 2 minutes at 95°C, followed by 40 cycles of 15 seconds at 95°C and 30 seconds at 60°C.

The EPclin score, which combines the EP score with two clinical risk factors (nodal status and tumor size), was calculated as previously described [6]: EPclin score = 0.35∙*t* + 0.64∙*n* + 0.28∙*s*, where *t* codes the tumor size (1: ≤1 cm, 2: >1 to ≤2 cm, 3: >2 to ≤5 cm and 4: >5 cm), *n* codes the nodal status (1: negative, 2: one to three positive nodes, 3: four to ten positive nodes, and 4: more than ten positive nodes) and *s* is the EP score. Both EP and EPclin scores were used to stratify patients into low-risk and high-risk groups [6]. Patients with an EP score <5 (EPclin score <3.3) were classified as being at low risk for distant recurrence, and patients with an EP score ≥5 (EPclin score ≥3.3) were categorized as being at high risk.

### Statistical analysis

The primary endpoint of the present study was distant metastasis-free survival (MFS), defined as the interval between the date of randomization until the date of distant metastatic recurrence or death due to disease progression as the first event. Deaths due to any other causes were censored. We define *distant metastatic recurrence* as excluding ipsilateral breast tumor recurrence, regional invasive recurrence (ipsilateral axilla, internal mammary and infra- and supraclavicular node metastases), contralateral BC and all *in situ* carcinomas. Because these events are potentially nonlethal, they were also censored. Overall survival (OS) was the secondary endpoint. All analyses were conducted according to a prespecified statistical analysis plan using predefined objectives and cutoff values in accordance with the prospective-retrospective design outlined by Simon *et al*. [22]. Metastasis rates and OS were estimated using the Kaplan–Meier method. A logrank test was used to compare MFS and OS between EP risk groups and between treatment arms. We used Cox proportional hazards models to calculate hazard ratios (HRs) and their 95% confidence intervals (95% CIs) for all analyzed endpoints. Associations and interactions were assessed by using multivariate Cox proportional hazards models. Two-sided tests were used to determine *P-*values, and *P-*values <0.05 were considered statistically significant. Unbiased concordance statistics were estimated for common clinicopathological parameters (age; nodal status; tumor size; treatment arm; tumor grade; and ER and PR status and Ki67 index), EP/EPclin scores and combinations of them using cross-validation and resampling. We calculated *P*-values to test whether combinations of molecular parameters or clinical variables were significantly associated with distant metastases.

## Results

The results of our present study are presented in accordance with reporting recommendations for tumor marker prognostic studies criteria.

### Validation of the EP test in ER+/HER2− breast cancer patients from the GEICAM 9906 trial

As mentioned above, patients in the GEICAM 9906 trial were retrospectively analyzed to evaluate the prognostic performance of EP in node-positive, ER+/HER2−, chemotherapy-treated patients. A total of 566 of the 800 available tumor samples were categorized as ER+/HER2− by central gene expression assessment and therefore were considered eligible for EP measurement. EP was successfully determined in 555 (98%) of the 566 ER+/HER2− samples, whereas 11 samples (2%) were excluded from further analysis for technical reasons (see the CONSORT diagram in Additional file 2: Figure S2). Table 1 gives the patient characteristics, the demographic and prognostic features and the 7-year MFS and OS of patients whose tumor samples had been centrally tested and were found to be similar to those that had not been centrally tested (data not shown) [17]. The 555 eligible patients were assigned to one of two risk categories (low or high) according to the predefined EP cutoff value [6]. Twenty-five percent (*n* = 141) of the ER+/HER2− BC patients were classified as low risk according to EP score.

**Table 1:** **Characteristics of participating breast cancer patients with ER+/HER2− tumors**^**a**^

**Characteristics**	**Patients, *n *** **(%)**	**Measurement results**	**Patients, *n *** **(%)**
**Age, yr**		**ER (Allred score**^**b**^**)**	
<50	250 (45%)	0	53 (9.6%)
≥50	305 (55%)	3	5 (0.9%)
**Menopausal status**		4	14 (2.5%)
Premenopausal	300 (54%)	5	28 (5.1%)
Postmenopausal	255 (46%)	6	66 (11.9%)
**Nodal status**		7	130 (23.4%)
N1	357 (64%)	8	256 (46.1%)
N2	151 (27%)	Unknown	3 (0.5%)
N3	47 (9%)	**PR (Allred score**^**b**^**)**	
**T stage**		0	104 (18.7%)
1	252 (45%)	3	10 (1.8%)
2	276 (50%)	4	14 (2.5%)
3	27 (5%)	5	42 (7.6%)
**Grade**		6	48 (8.7%)
1	91 (16%)	7	65 (11.7%)
2	260 (47%)	8	268 (48.3%)
3	157 (28%)	Unknown	4 (0.7%)
Unknown	47 (9%)	**Ki67 (%)**	
		Median (min-max) = 5 (0 to 80)	
**Treatment arm**		Low (**<**14%)	400 (72.1%)
FEC	280 (50.5)	High (**≥**14%)	134 (24.1%)
FEC-P	275 (49.5)	Unknown	21 (3.8%)

^a^ER: Estrogen receptor; FEC: Fluorouracil, epirubicin and cyclophosphamide; FEC-P: Fluorouracil, epirubicin and cyclophosphamide followed by weekly paclitaxel; HER2: Human epidermal growth factor receptor 2; PR: Progesterone receptor. ^b^Allred *et al*. [18]. Patient data were drawn from the GEICAM 9906 trial (*n* = 555).

**Figure 1:**
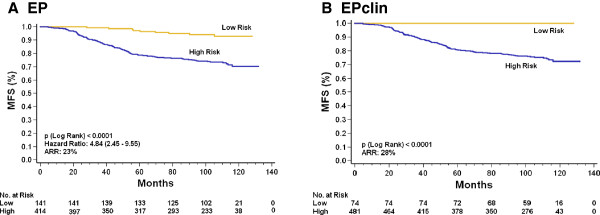
**Kaplan-Meier metastasis-free survival curves for ER+/HER2− breast cancers. (A)** Curves representing EndoPredict (EP) test results indicating estimated high and low risk of metastasis-free survival (MFS). The cutoff point was prespecified at 5. **(B)** Curves representing EPclin results indicating estimated high and low risk of MFS. The cutoff point was prespecified at 3.3. Numbers in parentheses indicate the 95% confidence intervals of the hazard ratios. ARR: Absolute risk reduction estimated at 10 years; ER+/HER2−: Estrogen receptor–positive/human epidermal growth factor receptor 2–negative. The MFS in the EP score–based low-risk category was 93% vs 70% in the EP score–based high-risk group. The MFS in the EPclin-based low-risk category was 100% vs 72% in the EPclin score–based high-risk group.

The estimated rates of MFS at 10 years were 93% for the EP score–based low-risk group (9 events in 141 patients) and 70% for the EP score–based high-risk group (110 events in 414 patients), with an absolute risk reduction of 23% (HR = 4.8, 95% CI = 2.5 to 9.6; *P* < 0.0001) (Figure 1A). EP score–based risk categorization was also significantly associated with OS (secondary endpoint) in the GEICAM 9906 ER+/HER2− cohort (HR = 3.9, 95% CI = 2.0 to 7.5; *P* < 0.0001) (Additional file 3: Figure S3A).

**Figure 2:**
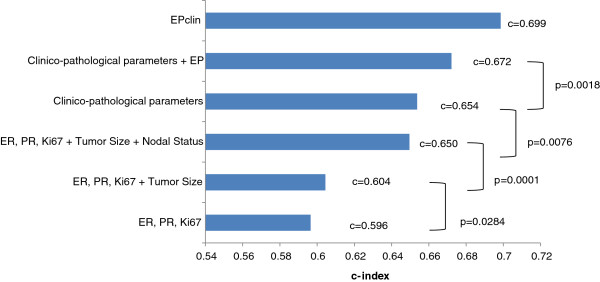
**C-index plot for clinicopathological parameters and EndoPredict test results between breast cancer patients with ER+/HER2− tumors.** C-index estimates for different groupings of prognostic parameters are shown: ER + PR + Ki67, ER + PR + Ki67 + tumor size, ER + PR + Ki67 + tumor size + nodal status, all clinicopathological parameters (nodal status, tumor size, grade, treatment arm, and ER and PR status and Ki67 index), all clinicopathological parameters + EndoPredict (EP) score, and combined molecular and clinical EPclin. ER: Estrogen receptor; PR: Progesterone receptor. *P-values* indicate whether additional molecular parameters add significant prognostic information to clinical variables.

After examining the univariate relationships between the clinicopathological variables and MFS (Table 2), on the basis of a multivariate Cox proportional hazards regression model adjusted for age, grade, nodal status, tumor size, treatment arm and ER and PR status and Ki67 index, we assessed whether EP could be used to provide independent prognostic information in the ER+/HER2− cohort. Nodal status and EP score remained significant in the multivariate model, suggesting that EP score is an independent predictor of distant metastasis (Table 3). In both Tables 2 and 3, the first *P*-value for each variable represents the level of statistical significance of the overall effect of each variable on MFS at the univariate and multivariate levels, respectively. Additionally, unbiased concordance index (C-index) estimates were calculated for the different combinations of the same clinicopathological parameters (age, grade, nodal status, tumor size, treatment arm and ER and PR status and Ki67 index), as well as EP score combined with clinical variables and EPclin score (see Figure 2), to evaluate their differential contributions to prognostic classification in ER+/HER2−, node-positive BC patients (C-index *P*-values were <0.05 in all cases; data not shown). The combination of all of the aforementioned clinicopathological parameters resulted in a C-index estimate of 0.65. The addition of the EP score to the combination of the clinicopathological markers resulted in a significant improvement in the predictive accuracy (*P* < 0.0018) and a C-index estimate of 0.67 (Figure 2). The EPclin score had the highest C-index estimate (0.70) in comparison to all analyzed combination strategies.

**Table 2:** Univariate Cox proportional hazards ratios and 95% confidence intervals in univariate analyses for association between EndoPredict, clinicopathological variables and metastasis-free survival

**Variables**	**HR (95% CI**^**a**^**)**	***P-*** **value**
**EP**	1.207 (1.134 to 1.285)	<0.0001
**EPclin**	1.916 (1.625 to 2.259)	<0.0001
**Age**	0.977 (0.960 to 0.993)	0.0065
**Tumor size**		0.0159^b^
**≤1**	Reference value	
**>1 to ≤2 cm**	1.038 (0.457 to 2.357)	0.9290
**>2 to ≤5 cm**	1.919 (0.884 to 4.168)	0.0994
**>5 cm**	2.102 (0.762 to 5.798)	0.1511
**Number of positive nodes**		<0.0001^b^
**1 to 3**	Reference value	
**4 to 10**	1.631 (1.085 to 2.451)	0.0187
**>10**	4.911 (3.022 to 7.979)	<0.0001
**Grade**		0.0233^b^
**G1**	Reference value	
**G2**	2.263 (1.157 to 4.428)	0.0171
**G3**	2.883 (1.446 to 5.746)	0.0026
**Unknown**	1.844 (0.749 to 4.538)	0.1829
**Treatment arm**		0.6067^b^
**FEC**	Reference value	
**FEC-P**	0.910 (0.635 to 1.304)	
**ER (Allred score**^**c**^**)**	0.950 (0.886 to 1.019)	0.1505
**PR (Allred score**^**c**^**)**	0.923 (0.874 to 0.974)	0.0033
**Ki67 (quantitative)**	1.017 (1.004 to 1.030)	0.0080

^a^EP: EndoPredict; EPclin: Combined molecular and clinical score; CI: Confidence interval; ER: Estrogen receptor; HR: Hazard ratio; MFS: Metastasis-free survival; PR: Progesterone receptor. Number of patients included in the analyses is 555. ^b^*P*-value of the variable’s overall effect on MFS. ^c^Allred *et al*. [18].

**Table 3:** Multivariate Cox proportional hazards ratios and 95% confidence intervals for the association between EndoPredict, selected clinicopathological variables and metastasis-free survival

**Variables**	**HR (95% CI**^**a**^**)**	***P-*** **value**
**EP**	1.126 (1.041 to 1.219)	0.0031
**Number Positive Nodes**		<0.0001^b^
**1 to 3**	Reference value	
**4 to 10**	1.420 (0.932 to 2.166)	0.1030
**>10**	3.605 (2.102 to 6.185)	<0.0001
**Age**	0.983 (0.966 to 1.001)	0.0628
**Tumor size**		0.6631^b^
**≤1 cm**	Reference value	
**>1 to ≤2 cm**	0.789 (0.343 to 1.816)	0.5774
**>2 to ≤5 cm**	1.042 (0.466 to 2.331)	0.9196
**>5 cm**	0.880 (0.301 to 2.577)	0.8159
**Treatment arm**		0.9331^b^
**FEC**	Reference value	
**FEC-P**	1.016 (0.697 to 1.482)	
**Grade**		0.4650^b^
**G1**	Reference value	
**G2**	1.662 (0.830 to 3.329)	0.1519
**G3**	1.589 (0.747 to 3.377)	0.2290
**Unknown**	1.198 (0.470 to 3.052)	0.7051
**ER (Allred score**^**c**^**)**	0.980 (0.903 to 1.063)	0.6207
**PR (Allred score**^**c**^**)**	0.965 (0.902 to 1.032)	0.2947
**Ki67 quantitative**	1.001 (0.986 to 1.016)	0.8982

^a^CI: Confidence interval; EP: EndoPredict; EPclin: Combined molecular and clinical score; ER: Estrogen receptor; HR: Hazard ratio; MFS: Metastasis-free survival; PR: Progesterone receptor. ^b^*P*-value of the variable’s overall effect on MFS. ^c^Allred *et al*. [18]. Multivariate analyses included 534 patients and 116 events.

EPclin was also used to dichotomize patients into low- and high-risk categories according to the threshold established for the EP test. EPclin score–based risk stratification was significantly associated with the distant metastasis rate in the GEICAM 9906 ER+/HER2− cohort. The estimated rates of MFS at 10 years were 100% for the EPclin-low risk group (0 events in 74 patients) and 72% for the EPclin high-risk group (119 events in 481 patients), with an absolute risk reduction of 28% (*P* < 0.0001) (Figure 1B). Interestingly, on the basis of EPclin test results, we identified a low-risk group (74 (13%) of 555 patients), which had no metastatic events and an OS rate of 99% (Additional file 3: Figure S3B).

### Prognostic performance in pre- and postmenopausal breast cancer patients

**Figure 3:**
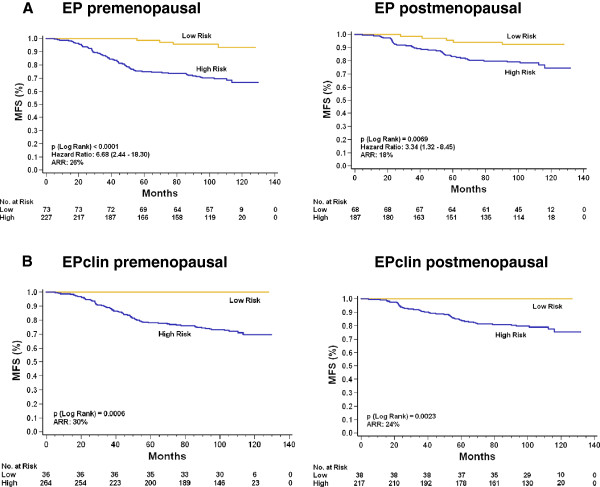
**Kaplan-Meier metastasis-free survival curves for ER+/HER2− breast cancers. (A)** Curves representing EndoPredict (EP) test results indicating estimated high and low risk of metastasis-free survival (MFS). The cutoff point for EP score–based risk stratification was prespecified at 5. For the premenopausal patients, MFS in the EP score–based low-risk category was 93% vs 67% in the EP score–based high-risk group. For the postmenopausal patients, MFS in the EP score–based low-risk category was 92% vs 74% in the EP score–based high-risk group. **(B)** Curves representing results based on the combined molecular and clinical EPclin indicating estimated high and low risk of MFS. The cutoff point for EPclin score–based risk stratification was prespecified at 3.3. In the EPclin premenopausal patients, MFS in the EPclin score–based low-risk group was 100% vs 70% in the EPclin score–based high-risk category. In the postmenopausal patients, MFS in the EPclin score–based low-risk category was 100% vs 76% in the EPclin score–based high-risk group. The samples included 300 premenopausal patients and 255 postmenopausal patients. Numbers in parentheses indicate the 95% confidence intervals of the hazard ratios. ARR: Absolute risk reduction estimated at 10 years.

The investigators in the GEICAM 9906 trial enrolled post- and premenopausal BC patients, allowing for subgroup analysis based on menopausal status. Of the 555 ER+/HER2− BC patients, 300 were premenopausal (54%) and 255 were postmenopausal (46%). In the subgroup analyses, 73 premenopausal patients (24.3%) and 68 postmenopausal patients (26.7%) were classified as being at low risk based on the EP test results. The subgroup analyses based on menopausal status suggested that EP is prognostic in ER+/HER2− BC patients in the premenopausal patients (HR = 6.7, 95% CI = 2.4 to 18.3; *P* < 0.0001) and postmenopausal patients (HR = 3.3, 95% CI = 1.3 to 8.5; *P* = 0.0069) (Figure 3A). The EPclin test results also generated a significant risk profile in premenopausal patients (*P* = 0.0006) and postmenopausal patients (*P* = 0.0023) with ER+/HER2− BC (Figure 3B).

### EndoPredict score and paclitaxel efficacy

**Figure 4:**
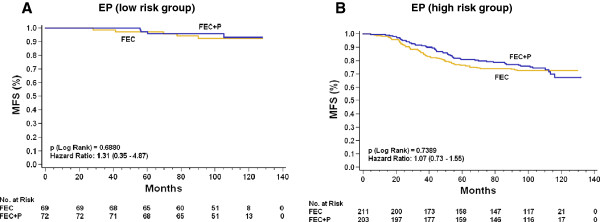
**Kaplan-Meier metastasis-free survival curves for ER+/HER2- breast cancer.** Comparison of treatment arms (FEC vs. FEC-P) in **(A)** the EP low-risk group (n = 141) and **(B)** the EP high-risk group (n = 414). The cutoff point for EP was prespecified at 5. Numbers in parentheses indicate the 95% confidence intervals of the hazard ratios. FEC: fluorouracil, epirubicin and cyclophosphamide; FEC+P: fluorouracil, epirubicin and cyclophosphamide followed by eight weekly courses of paclitaxel.

The GEICAM 9906 trial was conducted to compare the effect of adding weekly paclitaxel to conventional anthracycline-based (FEC) chemotherapy. Therefore, the treatment and outcome information produced in the randomized trial allowed us to test whether EP is predictive of whether treatment with weekly paclitaxel is greater efficacy than FEC. Adding weekly paclitaxel treatment did not significantly reduce the risk of relapse in the 555 ER+/HER2− BC patients analyzed (HR = 1.1, 95% CI = 0.8 to 1.6; *P* = 0.6067) (Additional file 4: Figure S4). MFS differences between treatment arms also failed to reach statistical significance in both the EP high- and low-risk groups (Figure 4). The interaction between the EP score and treatment arm was also nonsignificant (*P* = 0.71). Additionally, we found no significant treatment effects for OS (data not shown) or EPclin (Additional file 5: Figure S5). However, the 100% MFS of EPclin low-risk patients in the FEC arm suggests that this group might not benefit from the addition of paclitaxel.

## Discussion

The EP test has recently been validated in two large phase III trials (ABCSG6 and ABCSG8) that included postmenopausal ER+/HER2− BC patients treated with endocrine therapy alone. The ABCSG trials demonstrated that EP adds significant prognostic information to all commonly used clinicopathological parameters (including ER and Ki67) and clinical guidelines [6,13]. In line with the ABCSG6 and ABCSG8 clinical validation studies, we conducted a third clinical validation for the EP test by using archived tissue material according to the prospective-retrospective design described by Simon *et al*. [22]. In our present study, we analyzed EP-based data retrospectively in a large FFPE sample set derived from the phase III GEICAM 9906 trial on the basis of prospectively acquired clinical data and prespecified study objectives and laboratory assays.

To the best of our knowledge, this study is the first to show that EP is an independent prognostic parameter for both MFS and OS in node-positive, ER+/HER2− BC patients treated with adjuvant chemotherapy followed by hormone therapy. Also, in this cohort selected by ER+/HER2− status, EP test results add prognostic information to other common clinicopathological variables in this cohort. EP test results provide important information regarding the residual risk of recurrence after a modern anthracycline plus taxane chemotherapy regimen.

The results we report further suggest that EP better captures the tumor-derived intrinsic factors that lead to distant metastasis in node-positive, ER+/HER2− disease compared with some of the clinicopathological markers traditionally used to make treatment decisions (age, grade, nodal status, tumor size, treatment arm, and ER and PR status and Ki67 index).

What are the clinical implications of our results? The EP-/EPclin-based risk classification identifies a subgroup with a particularly low rate of distant metastatic events in a node-positive, high-risk cohort treated with anthracycline with or without a taxane-containing chemotherapy, followed by 5 years of endocrine therapy. In the face of 100% estimated distant MFS in the EPclin-based low-risk group, one might speculate that this patient group does not need an extension of endocrine therapy beyond 5 years. This finding is clinically relevant because the results of several clinical trials suggest that the extension of endocrine treatment should be considered in patients with ER+ BC in order to prevent late metastasis [23-28]. Additionally, EP-based low-risk patients can be sufficiently treated with standard chemotherapy. On the basis of our results, one might even speculate that the EP-based low-risk, node-positive ER+/HER2− patients treated with endocrine therapy might not derive any benefit from chemotherapy.

Several prognostic tests have been validated for BC patients in the past decade. The Onco*type* DX Breast Cancer Assay (Genomic Health, Redwood City, CA, USA) and the MammaPrint diagnostic test (Agendia, Irvine, CA, USA) were the first commercially available gene expression tests to predict the risk of recurrence in early-stage BC [1-3]. Whereas researchers in decision impact studies have demonstrated that both tests reduce health-care costs and spare patients from unnecessary chemotherapy [29-31]. Onco*type* DX has a higher level of supporting evidence than MammaPrint on the basis of large prospective-retrospective clinical validation studies. However, a recent biomarker substudy of the Arimidex, Tamoxifen Alone or in Combination trial (ATAC; ClinicalTrials.gov Identifier: NCT00849030) suggested that IHC4 provides prognostic information similar to that garnered from the Onco*type* DX assay [32]. Furthermore, IHC4 and the Clarient InsightDx Mammostrat test (Clarient Diagnostic Services, Aliso Viejo, CA, USA)—expanded immunohistochemical tests—can be used to determine the expression levels of diverse protein panels [33]. Although both tests are valuable as predictors for risk of recurrence, further statistical validation is still needed to ascertain whether these IHC tests can be standardized for everyday clinical use [34]. EP test results, together with other second-generation multigene tests (Predictor Analysis of Microarray 50-gene test (the PAM50 Breast Cancer Intrinsic Classifier) and Breast Cancer Index assay), have recently been introduced. In contrast to first-generation multigene algorithms and IHC4, these tests can also be used to predict late metastases [35-37]. Additionally, the PAM50 and EP tests can be carried out in a decentralized setting [15].

Most of the prognostic tests mentioned herein were initially developed to improve decision-making in the clinical management of node-negative BC patients [1,4,5,7]. Although some prognostic tests have been evaluated in heterogeneous patient populations, including patients with node-positive tumors, data regarding node-positive disease are scarce. Onco*type* DX was the first prognostic multigene test validated in a large clinical trial of node-positive patients, the SWOG-8814 study, which demonstrated that Onco*type* DX was effective in identifying subgroups with fair prognoses, although the likelihood of distant relapse still exceeded 30% in the low-risk group [9]. Our present study contributes to the literature by providing additional evidence suggesting that multigene tests can be used in patients with node-positive disease. Furthermore, in contrast to the Onco*type* DX–derived data mentioned above, our EP-based results strongly suggest that the EP-based low-risk group has a considerably low residual risk of distant recurrence (<10%) after standard chemotherapy. In contrast, high-risk patients have a high probability of exhibiting residual disease after conventional chemotherapy and should be considered for a tailored extension or intensification of adjuvant treatment, as well as for registration in clinical trials testing novel treatment strategies.

As a secondary aim in this study, we analyzed whether EP could be used to identify patients who might benefit from the addition of weekly paclitaxel to anthracycline-based chemotherapy treatment. Taxanes, although they are more toxic than conventional anthracycline-based regimens, are one of the most active cytotoxic agents and are widely used in standard chemotherapy. However, the absolute benefit of taxane-based treatment is small (3% to 7% absolute OS benefit within 5 years after treatment) [16,38-41] and needs to be balanced against serious side effects. Thus, there is an urgent need for novel predictors regarding which subgroups of patients stand to benefit substantially from treatment with taxanes. Of the several molecular markers analyzed as predictors of taxane efficacy [42-44], none of the markers or prognostic multigene assays have indicated that taxane benefits are maintained across relevant patient subgroups [10,11]. Similarly, our present results show that EP test results were not predictive of the efficacy of taxanes, because patients receiving weekly paclitaxel in addition to anthracycline-based chemotherapy did not experience any significant benefit. However, these results should be interpreted in the context of the main limitation of our study: a small sample size. Sample size is especially important in the validation of a predictive marker in patients with ER+/HER2− tumors, which are generally less chemoresponsive [45] and less effective in taxane-based treatment [16] compared with patients with other tumors.

The mechanistic determinants of taxane treatment response are poorly understood and still a matter of debate. Recently, we reported that a low proliferation score (11 cell-cycle genes) was predictive of weekly paclitaxel efficacy in the GEICAM 9906 trial [11]. Although the question whether this association also exists in patients with ER+/HER2− tumors was not examined, the association might be relevant in this context because proliferation is one of the strongest variables in calculating the EP test score, as well as in other available prognostic multigene tests. In contrast, the proliferation marker Ki67 was identified as predictive in luminal tumors treated with chemotherapy that included docetaxel [44], and patients with luminal B tumors benefited from taxane-based treatment [44,46]. The aforementioned conflicting results suggest that there are still a number of key processes in need of further study to elucidate the interaction between markers, taxane agents (paclitaxel and docetaxel), doses applied, administration schedules and treatment strategy duration.

## Conclusions

The results of our study show that EP provides additional prognostic information in the GEICAM 9906 trial ER+/HER2− cohort. This is the third clinical validation study of the EP score based on a prospective-retrospective design. It supports the high clinical evidence level of EP (level Ib according to Simon *et al*. [22]). Additionally, our study findings suggest that EP is prognostic in pre- and postmenopausal BC patients.

In line with other prognostic multigene assays, EP failed to predict the benefit of adding weekly paclitaxel to anthracycline-based chemotherapy in the GEICAM 9906 trial. However, EP might help to identify patients who are not being treated sufficiently with a standard taxane and/or anthracycline-based cytotoxic chemotherapy regimen and who might be eligible for treatment strategies with novel drugs.

## Abbreviations

BC: Breast cancer; CI: Confidence interval; EP: EndoPredict; ER: Estrogen receptor; FEC: Fluorouracil, epirubicin and cyclophosphamide; FEC-P: Fluorouracil, epirubicin and cyclophosphamide followed by weekly paclitaxel; FFPE: Formalin-fixed, paraffin-embedded; HER2: Human epidermal growth factor receptor 2; HR: Hazard ratio; MFS: Metastasis-free survival; RS: Recurrence score.

## Competing interests

JCB declares receiving salary from Sividon Diagnostics GmbH and holding a patent application related to the content of this article. KK and KF declare receiving salary from Sividon Diagnostics GmbH. KEW, RK and CP declare receiving salary from Sividon Diagnostics GmbH, holding shares in Sividon Diagnostics GmbH and holding a patent application related to the content of this article. The rest of the authors declare that they have no competing interests.

## Authors’ contributions

MM was involved in the conception and design of the study; acquisition, assembly analysis and interpretation of data; and drafting the manuscript. JCB participated in the conception and design of the study, designed the gene expression experiments, participated in the analysis and interpretation of data as well as statistical analysis and drafted the manuscript. KK designed and carried out the gene expression experiments, participated in the analysis and interpretation of data and was involved in drafting the manuscript. KF participated in the design of the study and in the statistical analysis and revised the manuscript critically. KEW participated in the design of the study and in the statistical analysis and revised the manuscript critically. RK participated in the conception and design of the study, participated in the analysis and interpretation of data and helped with drafting the manuscript. CP participated in the conception and design of the study, participated in the interpretation of data and helped with drafting the manuscript. LC, MRB, AR, BM, CR, CC, EA and ARL were involved in the acquisition and assembly of data and in the critical revision of the manuscript. MC completed statistical analyses and interpretation of data and critically revised the manuscript. EC and RC participated in the design and coordination of the study and helped with drafting the manuscript. All authors read and approved the final manuscript.

## Supplementary Material

Additional file 1: Figure S1 Participating centers in the GEICAM 9906 phase III clinical trial. Click here for file

Additional file 2: Figure S2 Diagram of the CONSORT study. Click here for file

Additional file 3: Figure S3 Kaplan-Meier overall survival curves for ER+/HER2− breast cancers by EndoPredict score and combined molecular and clinical EndoPredict test score risk groups. Cutoff points for EndoPredict (EP) and combined molecular and clinical EndoPredict test (EPclin) were prespecified at 5 and 3.3, respectively. Numbers in parentheses indicate the 95% confidence interval of the hazard ratio. EP: EndoPredict score. EPclin: combined molecular and clinical score. ARR: Absolute risk reduction estimated at 10 years. **(A)** Overall (OS) in EP low risk was 92% vs 67% in the EP high risk. **(B)** OS in EPclin low risk was 99% vs 69% in the EPclin high risk. Click here for file

Additional file 4: Figure S4 Kaplan-Meier metastasis-free survival curves for breast cancer patients with ER+/HER2− tumors. Analysis by treatment arm (FEC vs FEC-P). ER, Estrogen receptor; MFS, Metastasis-free survival. Click here for file

Additional file 5: Figure S5 Kaplan-Meier metastasis-free survival curves for ER+/HER2− tumors by treatment arm in EndoPredict clinical score **(A)** low-risk and **(B)** high-risk group. EPclin: combined molecular and clinical score. Cutoff point for EPclin was prespecified at 3.3. Numbers in parentheses indicate the 95% confidence interval of the hazard ratio. ER, Estrogen receptor; MFS, Metastasis-free survival. Click here for file
