# Supplementary material for: Clinical validation of the EndoPredict test in node-positive, chemotherapy-treated ER+/HER2− breast cancer patients: results from the GEICAM 9906 trial
Source: Breast Cancer Res. 2014 Apr 12;16(2):R38. doi: 10.1186/bcr3642 (PMC4076639; doi:10.1186/bcr3642)
Supplement: Additional file 1: Figure S1 — Participating centers in the GEICAM 9906 phase III clinical trial. [file bcr3642-S1.pdf]

PARTICIPATING CENTERS

- CENTRE HOSPITAL DE MANRESA, BARCELONA
- CENTRO ONCOLÓGICO DE GALICIA, A CORUÑA
- CLÍNICA ONCLOLÓGICA CORACHÁN, BARCELONA
- COMPLEJO HOSPITAL CIUDAD REAL, CIUDAD REAL
- COMPLEJO HOSPITAL ORENSE, ORENSE
- COMPLEJO HOSPITAL UNIVERSITARIO A CORUÑA, A CORUÑA
- COMPLEJO HOSPITALARIO DE JAÉN, JAÉN
- CONSORCI HOSPITAL PARC TAULÍ, BARCELONA
- CONSORCI SANITARI DE TERRASA, TARRASA
- FUNDACIÓN HOSPITAL ALCORCÓN, MADRID
- HOSPITAL ARNAU DE VILANOVA, VALENCIA
- HOSPITAL CLINIC I PROVINCIAL, BARCELONA
- HOSPITAL CLÍNICO UNIVERSITARIO DE VALENCIA, VALENCIA
- HOSPITAL CLÍNICO UNIVERSITARIO SAN CARLOS, MADRID
- HOSPITAL COMARCAL DE BARBASTRO, HUESCA
- HOSPITAL DE CABUEÑES, ASTURIAS
- HOSPITAL DE CRUCES, BILBAO
- HOSPITAL DE ELDA, ALICANTE
- HOSPITAL DE LA RIBERA, VALENCIA
- HOSPITAL DE LEÓN, LEÓN
- HOSPITAL DE NAVARRA, NAVARRA
- HOSPITAL DE TXAGORRITXU, VITORIA
- HOSPITAL DEL ESPÍRITU SANTO, BARCELONA
- HOSPITAL DEL MAR, BARCELONA
- HOSPITAL DONOSTIA, SAN SEBASTIÁN
- HOSPITAL DR. NEGRÍN, GRAN CANARIA
- HOSPITAL DR. PESET, VALENCIA
- HOSPITAL G. DE ALICANTE, ALICANTE
- HOSPITAL GENERAL DE ELCHE, ALICANTE
- HOSPITAL GENERAL DE GUADALAJARA, GUADALAJARA
- HOSPITAL GENERAL DE JEREZ, CÁDIZ
- HOSPITAL GENERAL DE MÓSTOLES, MADRID
- HOSPITAL GENERALUNIVERSITARIO DE VALENCIA, VALENCIA
- HOSPITAL GERMANS TRÍAS I PUJOL, BADALONA
- HOSPITAL INSULAR, GRAN CANARIA
- HOSPITAL JUAN RAMÓN JIMÉNEZ, HUELVA
- HOSPITAL LUCUS AUGUSTI, LUGO
- HOSPITAL MORALES MESSEGUER, MURCIA

- HOSPITAL MUNICIPAL DE BADALONA, BADALONA
- HOSPITAL MUTUA TERRASA, TARRASA
- HOSPITAL PROVINCIAL DE CÓRDOBA, CÓRDOBA
- HOSPITAL PROVINCIAL DE LA MISERICORDIA, TOLEDO
- HOSPITAL PROVINCIAL DE ZAMORA, ZAMORA
- HOSPITAL PUERTA DE HIERRO, MADRID
- HOSPITAL PUERTA DEL MAR, CÁDIZ
- HOSPITAL PUERTO DE SAGUNTO, VALENCIA
- HOSPITAL RAMÓN Y CAJAL, MADRID
- HOSPITAL RÍO CARRIÓN, PALENCIA
- HOSPITAL SANT JOAN DE REUS, TARRAGONA
- HOSPITAL UNIVERSITARIO DE CANARIAS, TENERIFE
- HOSPITAL UNIVERSITARIO DE SALAMANCA, SALAMANCA
- HOSPITAL UNIVERSITARIO DE VALLADOLID, VALLADOLID
- HOSPITAL UNIVERSITARIO LA FE, VALENCIA
- HOSPITAL UNIVERSITARIO MARQUÉS DE VALDECILLA, SANTANDER
- HOSPITAL UNIVERSITARIO MIGUEL SERVET, ZARAGOZA
- HOSPITAL UNIVERSITARIO PUERTO REAL, CÁDIZ
- HOSPITAL UNIVERSITARIO SAN CECLIO, GRANADA
- HOSPITAL UNIVERSITARIO VIRGEN DE LA ARRIXACA, MURCIA
- HOSPITAL UNIVERSITARIO VIRGEN DE LA VICTORIA, MÁLAGA
- HOSPITAL UNIVERSITARIO VIRGEN DEL ROCÍO, SEVILLA
- HOSPITAL VIRGEN DE LA SALUD, TOLEDO
- HOSPITAL VIRGEN DE LOS LIRIOS, ALICANTE
- HOSPITALCOMPLEJOUNIVERSITARIO LOZANO BLESA, ZARAGOZA
- INSTITUTO VALENCIANO DE ONCOLOGÍA, VALENCIA
- ONKOLOGIKOA, SAN SEBASTIÁN
