# Supplementary material for: Clinical validation of the EndoPredict test in node-positive, chemotherapy-treated ER+/HER2− breast cancer patients: results from the GEICAM 9906 trial
Source: Breast Cancer Res. 2014 Apr 12;16(2):R38. doi: 10.1186/bcr3642 (PMC4076639; doi:10.1186/bcr3642)
Supplement: Additional file 2: Figure S2 — Diagram of the CONSORT study. [file bcr3642-S2.pptx]

## Slide 1
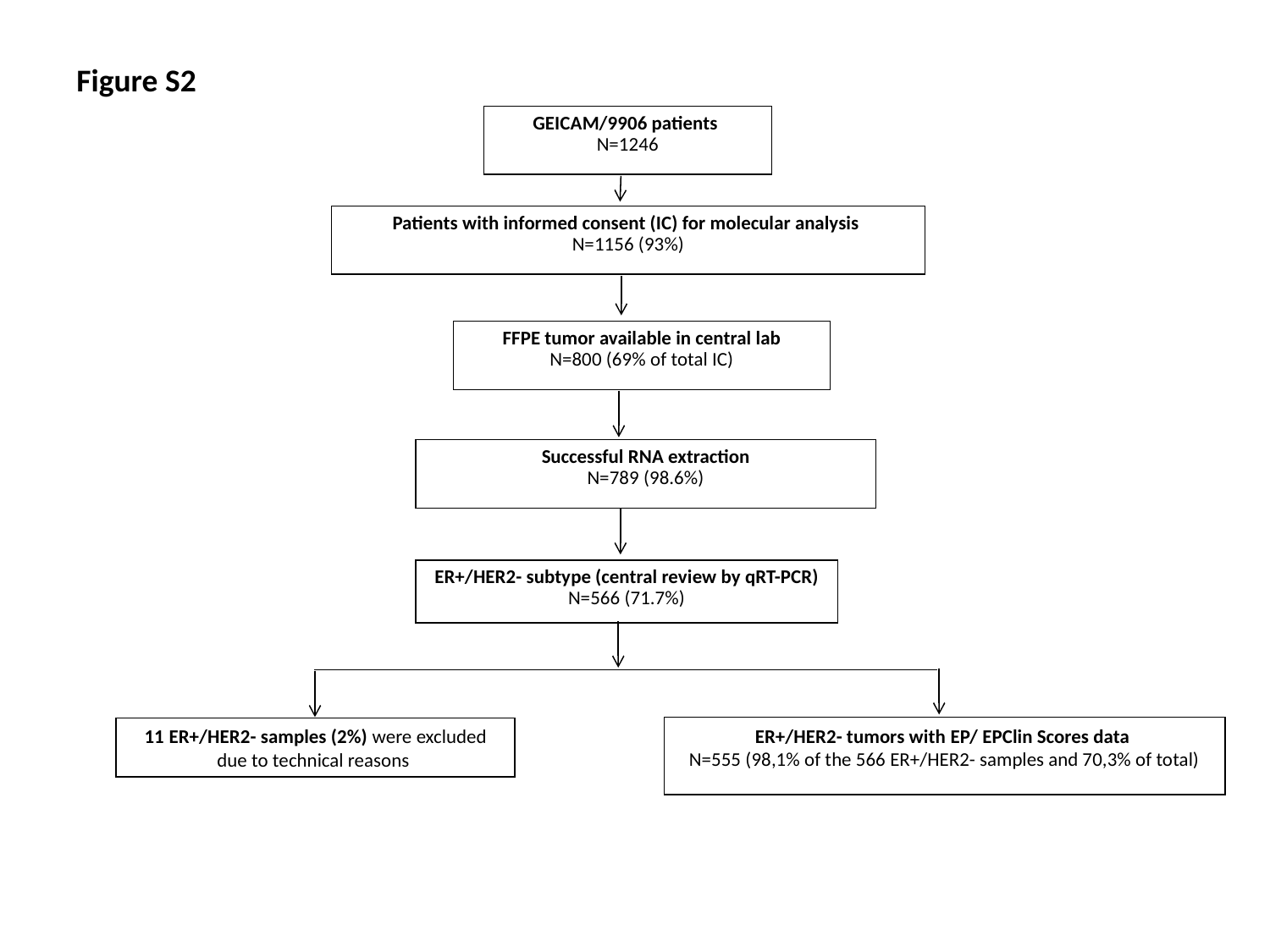

Figure S2
GEICAM/9906 patients
N=1246
Patients with informed consent (IC) for molecular analysis
N=1156 (93%)
FFPE tumor available in central lab
N=800 (69% of total IC)
Successful RNA extraction
N=789 (98.6%)
ER+/HER2- subtype (central review by qRT-PCR)
N=566 (71.7%)
ER+/HER2- tumors with EP/ EPClin Scores data
N=555 (98,1% of the 566 ER+/HER2- samples and 70,3% of total)
11 ER+/HER2- samples (2%) were excluded due to technical reasons
