# Supplementary figures and images for: Clinical validation of the EndoPredict test in node-positive, chemotherapy-treated ER+/HER2− breast cancer patients: results from the GEICAM 9906 trial
Source: Breast Cancer Res. 2014 Apr 12;16(2):R38. doi: 10.1186/bcr3642 (PMC4076639; doi:10.1186/bcr3642)

## Slide 1
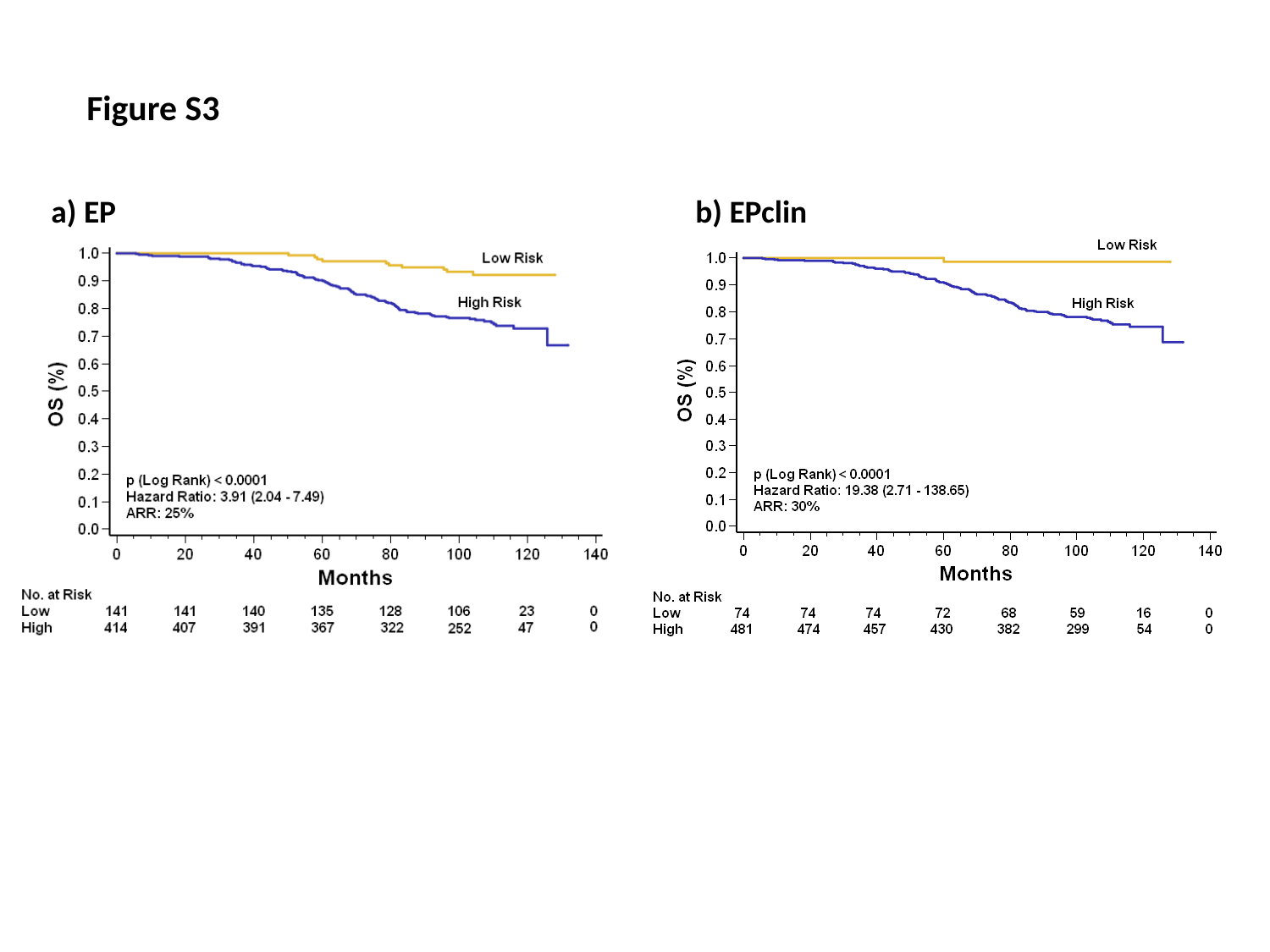

Figure S3
a) EP
b) EPclin

Supplement: Additional file 3: Figure S3 — Kaplan-Meier overall survival curves for ER+/HER2− breast cancers by EndoPredict score and combined molecular and clinical EndoPredict test score risk groups. Cutoff points for EndoPredict (EP) and combined molecular and clinical EndoPredict test (EPclin) were prespecified at 5 and 3.3, respectively. Numbers in parentheses indicate the 95% confidence interval of the hazard ratio. EP: EndoPredict score. EPclin: combined molecular and clinical score. ARR: Absolute risk reduction estimated at 10 years. (A) Overall (OS) in EP low risk was 92% vs 67% in the EP high risk. (B) OS in EPclin low risk was 99% vs 69% in the EPclin high risk. [file bcr3642-S3.pptx]

## Slide 1
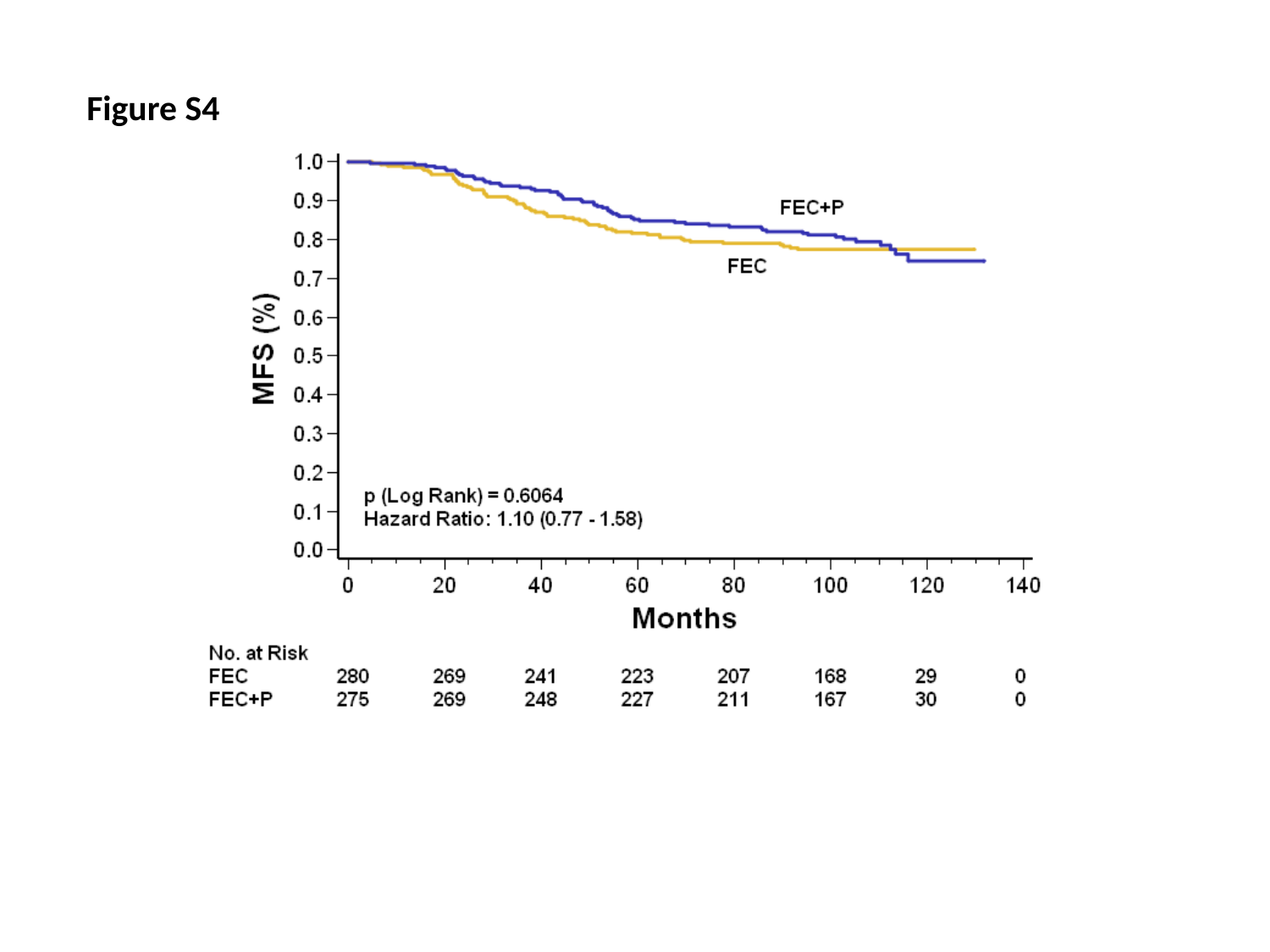

Figure S4

Supplement: Additional file 4: Figure S4 — Kaplan-Meier metastasis-free survival curves for breast cancer patients with ER+/HER2− tumors. Analysis by treatment arm (FEC vs FEC-P). ER, Estrogen receptor; MFS, Metastasis-free survival. [file bcr3642-S4.pptx]
