# Supplementary material for: Clinical validation of the EndoPredict test in node-positive, chemotherapy-treated ER+/HER2− breast cancer patients: results from the GEICAM 9906 trial
Source: Breast Cancer Res. 2014 Apr 12;16(2):R38. doi: 10.1186/bcr3642 (PMC4076639; doi:10.1186/bcr3642)
Supplement: Additional file 5: Figure S5 — Kaplan-Meier metastasis-free survival curves for ER+/HER2− tumors by treatment arm in EndoPredict clinical score (A) low-risk and (B) high-risk group. EPclin: combined molecular and clinical score. Cutoff point for EPclin was prespecified at 3.3. Numbers in parentheses indicate the 95% confidence interval of the hazard ratio. ER, Estrogen receptor; MFS, Metastasis-free survival. [file bcr3642-S5.pptx]

## Slide 1
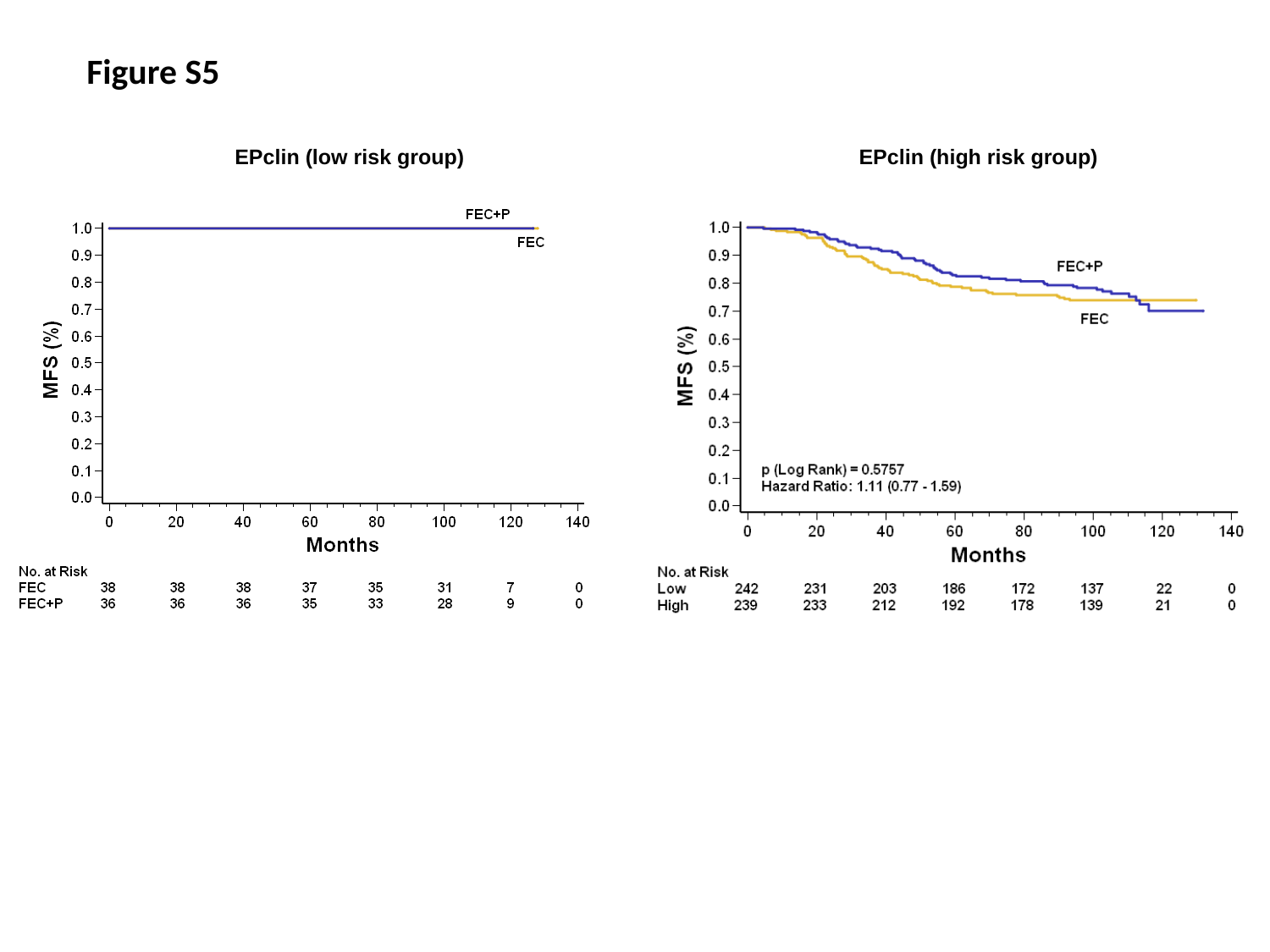

Figure S5
EPclin (low risk group)
EPclin (high risk group)
No distant metastases in the EPclin low-risk group
